# Cysteine Proteases as Validated Targets for the Treatment of Neglected and Poverty-Related Parasitic Diseases

**DOI:** 10.3390/ijms241210097

**Published:** 2023-06-13

**Authors:** Roberta Ettari

**Affiliations:** Department of Chemical, Biological, Pharmaceutical and Environmental Sciences, University of Messina, Viale Ferdinando Stagno d’Alcontres 31, 98166 Messina, Italy; rettari@unime.it; Tel.: +39-090-676-6554

Neglected tropical diseases (NTDs) include 20 diverse infections mainly prevalent in tropical areas that mostly affect disadvantaged communities and women and children. These diseases cause serious health problems and social and economic consequences to more than one billion people. The epidemiology of NTDs is very complex since they are strictly dependent on environmental conditions. Many are vector-borne and have animal reservoirs; thus, their control is a very difficult challenge [1].

Human African Trypanosomiasis (HAT or sleeping sickness) is an NTD caused by two subspecies of protozoa belonging to the *Trypanosoma* genus: *T. brucei gambiense* and *T. brucei rhodesiense. T. b. gambiense* is widespread in central and western Africa and causes a chronic form of the disease, while *T. b. rhodesiense* is most common in southern and eastern Africa and causes an acute form of trypanosomiasis with a high mortality rate [2]. Current HAT therapy is based on a few dated drugs characterized by toxicity problems, a limited spectrum of action, and parenteral administration [3,4]. Nifurtimox-eflornithine combination therapy (NECT) is the current first-line therapy for the treatment of the gambiense form of HAT [5]. Even the most recent agent, fexinidazole, a new 5-nitroimidazole derivative introduced in therapy for oral administration, was approved only for the gambiense form of HAT [6].

In this context, rhodesain and TbCatB, the cathepsin L-like (TbCatL) and cathepsin B-like (TbCatB) cysteine proteases of *T. brucei,* respectively, could represent promising targets for HAT treatment [7,8].

Both cathepsin L and B share the common feature of Clan CA cysteine proteases, including the conserved catalytic triad (Cys/His/Asn); the protease also adopts the typical papain-like folding: it is composed of two domains, left (L) and right (R), with the catalytic triad located in a cleft between the two domains [9].

Rhodesain and TbCatB are the sole Clan CA papain-like cysteine proteases produced by the Trypanosoma during infection of the mammalian host and are involved in the progression of the disease.

Rhodesain is important due to its several functions: it is required to cross the blood–brain barrier (BBB), thus causing the neurological stage of the disease [10]; this occurs by the activation of Gq protein-coupled receptors, also known as protease-activated receptors (PARs), whose activation increases intracellular calcium levels, leading to cytoskeletal changes in endothelial cells and BBB dysfunction [11]. Rhodesain is also involved in the turnover of the variant surface glycoprotein (VSG) of the Trypanosoma coat, thus allowing the parasite to evade the host immune system [12]. The trypanosomal cysteine protease also shows a relevant proteolytic activity in the lysosome, being involved in the degradation of parasite proteins and intracellularly transported host proteins; its involvement in the degradation of host immunoglobulins has also been highlighted [13]. On the other hand, TbCatB was demonstrated to be required for the degradation of transferrin. The bloodstream form (BSF) of *T. brucei* acquires iron from the host by internalizing transferrin by receptor-mediated endocytosis; the transferrin is then promptly degraded in the parasite lysosome through TbCatB [14]. First, RNA interference studies showed that the silencing of TbCatB cured infected mice, whereas TbCatL suppression only prolonged the lifespan of the infected mice, preventing the parasites from crossing the BBB and reaching the central nervous system (CNS) [15]. However, more recent studies gave a re-interpretation of the relative roles of TbCatB and TbCatL for the life cycle of *T. brucei*, suggesting that TbCatL is the essential cysteine protease for the survival of *T. brucei*. However, considering the different roles of rhodesain and TbCatB, inhibiting both enzymes is strongly desirable since it could block parasite replication during different phases and, consequently, host invasion [16].

Chagas disease is another NTD endemic in twenty-one Latin American countries due to the protozoan parasite *Trypanosoma cruzi*. The disease is characterized by an acute asymptomatic phase; then, in 30–40% of the patients, the chronic form is observed, with cardiac and gastrointestinal manifestations [17].

Current Chagas disease treatment is based on two nitro-substituted heterocyclic drugs, benznidazole and nifurtimox. However, both suffer from relevant drawbacks: they are associated with severe adverse effects, require prolonged treatments, and have no efficacy in the chronic phase of the disease [17]. Therefore, in this present scenario, it is of utmost importance to identify molecular targets to develop novel effective antitrypanosomal agents.

Cruzain (TcrCATL), also known as cruzipain (Cz), is the major cysteine protease of *Trypanosoma.* Cz is expressed by all forms and strains of the parasite, and it is secreted and can also be found in the parasite membrane. It has been demonstrated that the parasite’s virulence and morphogenesis strictly depend on the endogenous activity of Cz. In addition, Cz is fundamental for amastigote replication and plays a key role in host–parasite interactions; it stimulates the humoral and cellular immune responses during the infection. Thus, targeting Cz is currently considered a validated strategy for treating Chagas disease [18,19].

On the other hand, malaria, a poverty-related parasitic disease, is the most widespread and severe tropical infectious disease; in humans, it is caused by several species of the *Plasmodium* genus, with *Plasmodium falciparum* being the most dangerous and most prevalent species [20].

Current treatment of uncomplicated malaria caused by *P. falciparum* is based on artemisinin-based combination (ACT) therapies, which are built on the use of two antimalarial agents endowed with a different mechanism of action, one of which is an artemisinin derivative. However, the partial resistance to artemisinin was recently demonstrated to involve delayed parasite clearance [21].

In the present scenario, identifying novel targets to develop effective antimalarial agents is an urgent priority. In this context, falcipain-2 (FP-2) and falcipain-3 (FP-3) are considered promising targets for the development of novel antimalarial agents [22,23].

The primary role of FP-2 and FP-3 includes the degradation of host hemoglobin required for sustaining the metabolic needs of the rapidly growing parasite. They show a high level of sequence homology but different substrate specificity; FP-2 and FP-3 are primarily localized in the food vacuole, where they are involved in hemoglobin digestion. Both FPs are synthesized over long intervals during the erythrocytic cycle as membrane-bound proforms, and are then processed to mature forms. However, FP-2 is synthesized earlier, in the early trophozoites, and is processed more quickly than FP-3, which is expressed in the late trophozoites/early schizonts stage. During the late trophozoite and schizont stages, FP-2 is also involved in the degradation of erythrocyte membrane skeletal proteins, including ankyrin and the band 4.1 protein, thus contributing to the destabilization of the erythrocyte membrane, leading to host cell rupture and the release of the mature merozoites [24,25,26,27,28].

Several rhodesain and TbCatB inhibitors have been reported for HAT treatment [7,8], as well as several Cz inhibitors for Chagas disease [29]. Lastly, FP-2 and FP-3 inhibitors were proven to be potent agents for malaria treatment [22,23].

Generally, they differ for the type of inhibition, which is reversible or irreversible, depending on the warhead, with the most potent inhibitors demonstrating activity against both the target cysteine protease and the related parasite, thus giving hope that these inhibitors can further progress into clinical trials to find new effective and broad-spectrum drugs for NTDs or poverty-related parasitic diseases.
